# Analysis of Collection of Hemolytic Uremic Syndrome–associated Enterohemorrhagic *Escherichia coli*

**DOI:** 10.3201/eid1408.071082

**Published:** 2008-08

**Authors:** Alexander Mellmann, Martina Bielaszewska, Robin Köck, Alexander W. Friedrich, Angelika Fruth, Barbara Middendorf, Dag Harmsen, M. Alexander Schmidt, Helge Karch

**Affiliations:** * Institute for Hygiene and the National Consulting Laboratory on Hemolytic Uremic Syndrome, Münster, Germany; † Robert Koch Institute, Wernigerode, Germany; ‡ Periodontology, Münster; § Center for Molecular Biology of Inflammation, Münster

**Keywords:** Enterohemorrhagic *Escherichia coli*, EHEC, hemolytic uremic syndrome, HUS, HUSEC, serotyping, molecular phylogeny, MLST, dispatch

## Abstract

Multilocus sequence typing of 169 non-O157 enterohemorrhagic *Escherichia coli* (EHEC) isolated from patients with hemolytic uremic syndrome (HUS) demonstrated 29 different sequence types (STs); 78.1% of these strains clustered in 5 STs. From all STs and serotypes identified, we established a reference panel of EHEC associated with HUS (HUSEC collection).

Enterohemorrhagic *Escherichia coli* (EHEC) strains are a highly pathogenic subgroup of Shiga toxin–producing *E. coli* (STEC) that cause severe human diseases, including bloody diarrhea and hemolytic uremic syndrome (HUS) (*1*). The ability to cause severe human disease differentiates EHEC from other STEC found in the environment that are less pathogenic or nonpathogenic. *E. coli* O157:H7 is the most frequent EHEC implicated as a cause of HUS (*2*), but non–O157:H7 EHEC are variably present as the only pathogens in stools from HUS patients (*1*,*3*,*4*).

A recent phylogenetic analysis of *E. coli* isolated from humans and animals in different geographic areas by multilocus sequence typing (MLST), the current standard for phylogenetic analyses of bacteria, indicated extensive allelic variations and homolog recombinations in pathogenic lineages and demonstrated repeated and independent evolution of pathogenic strains (*5*,*6*). However, only a limited number of EHEC associated with HUS have been so investigated. Therefore, we performed a comprehensive MLST-based examination of the molecular phylogeny of EHEC isolated from HUS patients and established a collection of representative HUS-associated enterohemorrhagic *E. coli* (HUSEC) (www.ehec.org).

## The Study

From 1996 through 2006, 524 EHEC were isolated as the only pathogens from fecal samples of epidemiologically unrelated patients with HUS (1 strain per patient). The isolation was achieved by using previously described procedures (*7*). The isolates were confirmed as *E. coli* by API 20 E (bioMérieux, Marcy l’Etoile, France) and serotyped (*8*) by using antisera against *E. coli* O antigens 1–181 and H antigens 1–56. In all nonmotile isolates from serogroups O26, O103, O111, O145, and O157, *fliC* genes were genotyped (*9*,*10*). MLST was performed as described previously (*6*) with small modifications (*11*). Phylogenetic analyses were based on allelic data that used the BURST algorithm (*12*) to achieve a more robust interpretation of the clustering and to reduce the influences by the effects of the recombination, which are widespread in *E. coli* (*6*). In addition, the stringent definition of clonal complexes (CCs), with which strains sharing at least 6 identical alleles are grouped into the same CC, was applied. The minimum spanning tree was generated from the allelic profiles by using *Shigella dysenteriae* strain M1354 (ST243, by using data from http://web.mpiib-berlin.mpg.de/mlst/dbs/Ecoli) as outgroup ( Appendix Figure).

Among 524 EHEC isolated from HUS patients, 355 (67.7%) belonged to serotypes O157:H7/H^–^ (249 were non–sorbitol-fermenting EHEC O157:H7/H^–^ and 106 were sorbitol-fermenting EHEC O157:H^–^), and 169 (32.3%) belonged to 34 non-O157 serotypes. Because the phylogeny of *E. coli* O157:H7/ H^-^ has been extensively studied and is well established (*5*,*13*), we focused on MLST analysis of the 169 non-O157 strains, which represent all non-O157 EHEC serotypes from German HUS patients during the study period. We performed MLST analysis of only a subset of 10 O157 strains as controls.

MLST analysis of 169 non-O157 EHEC isolates distinguished 29 different sequence types (STs), which clustered into 10 CCs and 12 singletons (Table 1). The predominant ST was ST21, which consisted of 43 isolates (25.4% of non-O157 EHEC), followed by ST29 (30 isolates, 17.8%), ST32 (30 isolates, 17.8%), ST17 (15 isolates, 8.9%), and ST16 (14 isolates, 8.3%) (Table 1). These 5 STs included 78.1% of all HUS-associated non-O157 EHEC. The remaining 21.9% (n = 37) of the non-O157 EHEC strains belonged to 24 other STs that comprised only 1–3 strains; 14 of these STs were found only once (Table 1). Among the 10 CCs identified, CC29 was the most frequent. It comprised 89 strains of 5 STs, corresponding to 60.1% of 148 strains that could be assigned to a CC and to 52.7% of all non-O157 EHEC (Table 1). After CC29, CC32 and CC20 were the most frequently identified CCs (Table 1).

**Table 1:** Clonal complexes, sequence types, and serotypes of non-O157 EHEC from patients with hemolytic uremic syndrome*

CC	No. strains (%)†	ST	No. strains (%)†	Serotype‡ (no. strains)§
29	89 (52.7)	ST21	43 (25.4)	O26:H11/H^–^ (*fliC*_H11_) (41), OR:H11 (1), Ont:Hnt (1)
		ST29	30 (17.8)	O26:H11/H^–^ (*fliC*_H11_) (29), OR:H11 (1)
		ST27	1 (0.6)	O26:H11
		ST396	1 (0.6)	O26:H11
		ST16	14 (8.3)	O111:H8/H^–^ (*fliC*_H8_)
32	32 (18.9)	ST32	30 (17.8)	O145:H28/H^–^ (*fliC*_H28_)
		ST137	2 (1.2)	O145:H^–^ (*fliC*_H28_)
20	16 (9.5)	ST17	15 (8.9)	O103:H2/H^–^ (*fliC*_H2_) (14), OR:H2 (1)
		ST20	1 (0.6)	O119:H2
10	3 (1.8)	ST43	1 (0.6)	O111:H10
		ST330	2 (1.2)	Ont:H^–^
11	3 (1.8)	ST335	3 (1.8)	O55:H7
40	1 (0.6)	ST40	1 (0.6)	O112:H^–^
69	1 (0.6)	ST69	1 (0.6)	O73:H18
101	1 (0.6)	ST101	1 (0.6)	O55:Hnt
155	1 (0.6)	ST56	1 (0.6)	O113:H21
469	1 (0.6)	ST679	1 (0.6)	O163:H19
NA	2 (1.2)	ST25	2 (1.2)	O128:H2
NA	2 (1.2)	ST678	2 (1.2)	O104:H4
NA	2 (1.2)	ST655	2 (1.2)	O121:H19
NA	1 (0.6)	ST329	1 (0.6)	O136:Hnt
NA	3 (1.8)	ST342	2 (1.2)	O145:H25/H^–^ (*fliC*_H25_)
		ST659¶	1 (0.6)	O145:H^–^ (*fliC*_H25_)
NA	1 (0.6)	ST677	1 (0.6)	O174:H21
NA	1 (0.6)	ST39	1 (0.6)	O70:H8
NA	1 (0.6)	ST675	1 (0.6)	O76:H19
NA	3 (1.8)	ST442	3 (1.8)	O91:H21
NA	3 (1.8)	ST306	3 (1.8)	O98:H^–^ (2), OR:H^–^(1)
NA	2 (0.6)	ST672	2 (1.2)	O104:H21 (1), Ont:H21 (1)

*CC, clonal complex; ST, sequence type; EHEC, enterohemorrhagic *Escherichia coli*; HUS, hemolytic uremic syndrome; NA, not assigned. †% of strains of a CC and ST among all 169 non-O157 EHEC isolated from HUS patients. ‡H^–^, nonmotile; OR, O rough (autoagglutinable strain); nt, not typeable by the *E. coli* O and H antisera used. §Number of strains of the serotype that belonged to the respective ST; if no number is given, all strains of the serotype belonged to the respective ST. ¶ST659 is a single-locus variant of ST342.

The predominant serotypes identified among the 169 non–O157 HUS-associated EHEC were O26:H11/H– (n = 72; 42.6%), O145:H28/H– (n = 32; 18.9%), O111:H8/H– (n = 14; 8.3%), and O103:H2/H^–^ (n = 14; 8.3%). The nonmotile strains within these serogroups shared the H antigen–encoding *fliC* gene with the motile strains that expressed the respective H antigen (Table 1). These 8 serotypes together constituted 132 (78.1%) of the non-O157 EHEC associated with HUS, whereas the other 37 strains (21.9%) belonged to 26 different serotypes, 17 of which contained only a single isolate (Table 1).

The most frequent serotypes including O26:H11/H–, O103:H2/H–, O111:H8/H–, and O145:H28/H– clustered into the 5 most prevalent STs (Table 1). However, not all isolates of the same serotype always belonged to the same ST (Table 1). One example is serotype O26:H11/H^–^ (*fliC*_H11_), which was the most common non–O157 EHEC associated with HUS and clustered into 4 STs as single-locus variants (Table 1). Each of four O rough (OR) strains (2 OR:H11, and 1 each OR:H2 and OR:H^–^), none of which could be successfully serotyped, was matched by its ST to an O typeable strain, indicating a recent conversion from the smooth to the rough strain form.

The relationships among members of the different STs and CCs are demonstrated in the Appendix Figure. Within the serogroup O111, 14 isolates belonging to serotypes O111:H8 and O111:H^–^ (*fliC*_H8_) were ST16 (CC29). In contrast, the EHEC O111:H10 isolate with ST43 (CC10) shared none of the 7 MLST loci with the O111:H8/H^–^ strains, indicating that EHEC O111 causing HUS originate from 2 different clonal sources. Similar differences were observed between EHEC O145:H25 (ST342)/O145:H^–^ (*fliC*_H25_) (ST659) and O145:H28 (ST32). Whereas ST659 is a single-locus variant of ST342, both allelic profiles differ in all loci from ST32.

The combination of MLST analysis and serotyping enabled us to establish the HUSEC collection. This collection comprises 41 EHEC isolated from HUS patients in Germany, which includes all 36 EHEC serotypes (O157 and non-O157) isolated from HUS patients and all 31 STs identified within these serotypes (Table 2). The strains included in this HUSEC collection were reserotyped and characterized for their *stx* genotypes and the presence of the *eae* gene (Table 2). Phenotypic characteristics and additional properties such as putative virulence determinants are available at www.EHEC.org.

**Table 2:** Strains of the HUSEC collection representing all serotypes of HUS-associated EHEC strains isolated in Germany, 1996–2006*

Strain	Original	Year of isolation	Serotype	ST (CC)	*eae*	*stx*_1_	*stx*_2_†
HUSEC001	05-946	2005	O111:H10	43 (10)	–	–	2
HUSEC002	5152/97	1997	Ont:H^–^	330 (10)	–	–	2
HUSEC003	6334/96	1996	O157:H7	11 (11)	+	–	2
HUSEC004	3072/96	1996	O157:H^–^	11 (11)	+	–	2
HUSEC005	2907/97	1997	O55:H7	335 (11)	+	–	2
HUSEC006	5376/99	1999	O157:H^–^	587 (11)	+	–	2
HUSEC007	7382/96	1996	O103:H2	17 (20)	+	–	2
HUSEC008	2791/97	1997	O103:H^–^	17 (20)	+	–	2
HUSEC009	6833/96	1996	OR:H2	17 (20)	+	–	2
HUSEC010	1805/00/A	2000	O119:H2	20 (20)	+	1	–
HUSEC011	2516/00	2000	O111:H8	16 (29)	+	1	2
HUSEC012	6037/96	1996	O111:H^–^	16 (29)	+	1	2
HUSEC013	2245/98	1998	O26:H11	21 (29)	+	1	–
HUSEC014	5080/97	1997	O26:H^–^	21 (29)	+	1	2
HUSEC015	126814/98	1998	OR:H11	21 (29)	+	1	2
HUSEC016	5028/97	1997	Ont:Hnt	21 (29)	+	1	–
HUSEC017	3319/99	1999	O26:H11	27 (29)	+	1	2
HUSEC018	1530/99	1999	O26:H11	29 (29)	+	–	2
HUSEC019	1588/98	1998	OR:H11	29 (29)	+	1	–
HUSEC020	3271/00	2000	O26:H11	396 (29)	+	–	2
HUSEC021	0488/99	1999	O145:H28	32 (32)	+	–	2
HUSEC022	4557/99	1999	O145:H^–^	137 (32)	+	–	2
HUSEC023	1169/97/1	1997	O112:H^–^	40 (40)	–	–	2d_act_
HUSEC024	2996/96	1996	O73:H18	69 (69)	–	–	2d_act_
HUSEC025	06-05009	2006	O55:Hnt	101 (101)	–	1	–
HUSEC026	99-09355	1999	O113:H21	56 (155)	–	–	2d_act_
HUSEC027	03-07727	2003	O163:H19	679 (469)	–	–	2d_act_
HUSEC028	03-06687	2003	O128:H2	25 (NA)	–	1c	2d
HUSEC029	4256/99	1999	O70:H8	39 (NA)	+	–	2
HUSEC030	05-03519	2005	O98:H^–^	306 (NA)	–	1	–
HUSEC031	7792/96	1996	OR:H^–^	306 (NA)	+	1	–
HUSEC032	2441/98	1998	O136:Hnt	329 (NA)	–	1c	2
HUSEC033	4392/97	1997	O145:H25	342 (NA)	+	–	2
HUSEC034	3332/99	1999	O91:H21	442 (NA)	–	1	2+2d_act_
HUSEC035	1529/98	1998	O121:H19	655 (NA)	+	–	2
HUSEC036	2839/98	1998	O145:H^–^	659 (NA)	+	1	2c
HUSEC037	02-03885	2002	O104:H21	672 (NA)	–	1	2+2d_act_
HUSEC038	3356/97/B	1997	Ont:H21	672 (NA)	–	1	2d_act_
HUSEC039	3651/96	1996	O76:H19	675 (NA)	–	1c	–
HUSEC040	220/00	2000	O174:H21	677 (NA)	–	–	2c
HUSEC041	01-09591	2001	O104:H4	678 (NA)	–	–	2

*HUSEC, hemolytic uremic syndrome–associated enterohemorrhagic *Escherichia coli;* EHEC, enterohemorrhagic *E. coli*. For each serotype, the multilocus sequence type (ST) and the corresponding clonal complex (CC) are given in accordance to the *E. coli* multilocus sequence typing website (http://web.mpiib-berlin.mpg.de/mlst/dbs/Ecoli). Furthermore, the presence (+, present; – absent) of the intimin gene (*eae*), the Shiga toxin gene (*stx*), and its subtype(s) are specified. nt, not typeable by the O and H antisera used; H^–^, nonmotile; OR, O rough (autoagglutinable strain); NA, not assigned. †2d_act_, *stx_2_*_d-activatable_.

## Conclusions

Most (81.1%) of the non-O157 EHEC clustered into 3 CCs and belonged to a limited number of serotypes. These strains were recovered independently from different regions in Germany over an 11-year period. For the remaining strains, epidemiologic support is not as strong, and the clonal analysis demonstrated that their chromosomal backgrounds are highly divergent from those of CC29, CC32, and CC20. In 14 STs, we have only 1 isolate. In these cases, excluding concurrent or recent infection by *E. coli* O157 serologically is even more important. This exclusion was not always possible because patients’ serum for the investigation of immunoglobulin M anti-O157 lipopolysaccharide antibodies is frequently not available. However, at least some of these strains might represent emerging clones in the human population, such as O111:H10 (*10*), O113:H21 (*14*), and O121:H19 (*15*). Thus, strains of these serotypes included in our HUSEC collection can be used in future studies as a reference to compare EHEC isolated in other countries from HUS patients. This would allow timely discovery of the emergence of new non-O157 clones associated with HUS and the virulence traits that they contain (www.ehec.org).

## Supplementary Material

Appendix Figure  Minimum spanning tree of hemolytic uremic syndrome–associated enterohemorrhagic *Escherichia coli* strains and *Shigella dysenteriae* M1354 (ST243, data from the *E. coli* multilocus sequence type [ST] website [http://web.mpiib-berlin.mpg.de/mlst/dbs/Ecoli]) as an outgroup generated from allelic profiles based on the eBURST algorithm (*12)*. Each ST is represented by a circle named with its ST, and the corresponding serotypes are given (OR, O rough; H^–^, nonmotile; nt, not typeable with the *E. coli* O and H antisera used). Black lines connecting pairs of STs indicate that they share 6 (thick lines) or 5 (thin) alleles. Gray lines connecting pairs of STs of increasing line length indicate that the STs share <4 alleles. In addition, the STs and, if applicable, the connecting lines of a clonal complex are shaded in gray.
