# Supplementary figures and images for: Analysis of Collection of Hemolytic Uremic Syndrome–associated Enterohemorrhagic Escherichia coli
Source: Emerg Infect Dis. 2008 Aug;14(8):1287–90. doi: 10.3201/eid1408.071082 (PMC2600372; doi:10.3201/eid1408.071082)

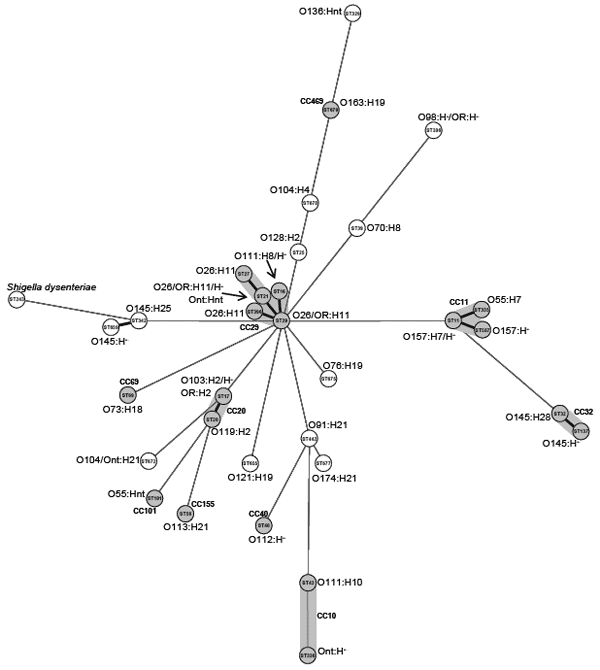

Supplement: Appendix Figure — Minimum spanning tree of hemolytic uremic syndrome–associated enterohemorrhagic Escherichia coli strains and Shigella dysenteriae M1354 (ST243, data from the E. coli multilocus sequence type [ST] website [http://web.mpiib-berlin.mpg.de/mlst/dbs/Ecoli]) as an outgroup generated from allelic profiles based on the eBURST algorithm (12). Each ST is represented by a circle named with its ST, and the corresponding serotypes are given (OR, O rough; H–, nonmotile; nt, not typeable with the E. coli O and H antisera used). Black lines connecting pairs of STs indicate that they share 6 (thick lines) or 5 (thin) alleles. Gray lines connecting pairs of STs of increasing line length indicate that the STs share <4 alleles. In addition, the STs and, if applicable, the connecting lines of a clonal complex are shaded in gray. [file 07-1082_app-s1.gif]
